# Combination chemotherapy with intermittent erlotinib and pemetrexed for pretreated patients with advanced non-small cell lung cancer: a phase I dose-finding study

**DOI:** 10.1186/1471-2407-12-296

**Published:** 2012-07-18

**Authors:** Seigo Minami, Takashi Kijima, Ryo Takahashi, Hiroshi Kida, Takeshi Nakatani, Masanari Hamaguchi, Yoshiko Takeuchi, Izumi Nagatomo, Suguru Yamamoto, Isao Tachibana, Kiyoshi Komuta, Ichiro Kawase

**Affiliations:** 1 Department of Respiratory Medicine, Osaka Police Hospital, 10-31 Kitayama-cho, Tennoji-ku, Osaka 543-0035, Japan; 2 Department of Respiratory Medicine, Allergy and Rheumatic Diseases, Osaka University Graduate School of Medicine, 2-2 Yamada-oka, Suita, Osaka 565-0871, Japan; 3 Present Affiliation: Osaka Prefectural Medical Center for Respiratory and Allergic Diseases, 3-7-1 Habikino, Habikino, Osaka 583-8588, Japan

**Keywords:** Combination, Erlotinib, Pemetrexed, NSCLC, Phase I

## Abstract

**Background:**

Erlotinib and pemetrexed have been approved for the second-line treatment of non-small cell lung cancer (NSCLC). These two agents have different mechanisms of action. Combined treatment with erlotinib and pemetrexed could potentially augment the antitumor activity of either agent alone. In the present study, we investigated the safety profile of combined administration of the two agents in pretreated NSCLC patients.

**Methods:**

A phase I dose-finding study (Trial registration: UMIN000002900) was performed in patients with stage III/IV nonsquamous NSCLC whose disease had progressed on or after receiving first-line chemotherapy. Patients received 500 mg/m^2^ of pemetrexed intravenously every 21 days and erlotinib (100 mg at Level 1 and 150 mg at Level 2) orally on days 2–16.

**Results:**

Twelve patients, nine males and three females, were recruited. Patient characteristics included a median age of 66 years (range, 48–78 years), stage IV disease (nine cases), adenocarcinoma (seven cases) and activating mutation-positives in the epidermal growth factor receptor gene (two cases). Treatment was well-tolerated, and the recommended dose of erlotinib was fixed at 150 mg. Dose-limiting toxicities were experienced in three patients and included: grade 3 elevation of serum alanine aminotransferase, repetitive grade 4 neutropenia that required reduction of the second dose of pemetrexed and grade 3 diarrhea. No patient experienced drug-induced interstitial lung disease. Three patients achieved a partial response and stable disease was maintained in five patients.

**Conclusions:**

Combination chemotherapy of intermittent erlotinib with pemetrexed was well-tolerated, with promising efficacy against pretreated advanced nonsquamous NSCLC.

## Background

Lung cancer is the leading cause of cancer-related death worldwide [1]. Non-small-cell lung cancer (NSCLC) accounts for approximately 85% of all lung cancer cases. Most of these cases are found to have locally advanced or systemically metastasized disease at the time of diagnosis. Currently, platinum-based chemotherapeutic regimens used in combination with one of the third-generation agents are recommended as the standard first-line treatment for advanced NSCLC, based on evidence of improved survival and quality of life (QOL) [2-5]. However, the efficacy of these first-line regimens has reached a plateau. Although currently docetaxel, pemetrexed and erlotinib have been approved for second-line treatment, only about 10% of patients respond to monotherapy using any of these agents [6-8]. To date, combination of cytotoxic drugs has also not been shown to provide a survival benefit in a second-line setting. Indeed, combination therapy causes excessive toxicities, which often make it difficult for patients to continue receiving treatment, irrespective of any additive antitumor effect.

Pemetrexed (Alimta®) is a multi-targeting antifolate cytotoxic agent that mainly inhibits three key enzymes involved in folate metabolism, namely thymidylate synthase, dihydrofolate reductase and glycinamide ribonucleotide formyl transferase [9]. When exposed to pemtrexed, a broad spectrum of solid cancer cells are arrested, mainly in the S-phase, and subsequently become apoptotic. A phase III trial in a second-line setting demonstrated that pemetrexed significantly improved tolerability, while maintaining an equivalent survival benefit as compared with docetaxel in pretreated NSCLC patients [7]. Because doses of pemetrexed as high as 1000 mg/m^2^ have not been found to be superior to a dose of 500 mg/m^2^ in terms of efficacy, the recommended dose (RD) has been fixed at this lower level [10,11]. Pemetrexed became available in Japan for the treatment of NSCLC at the approved dose of 500 mg/m^2^ in May 2009.

Erlotinib (Tarceva®), an oral and reversible epidermal growth factor receptor (EGFR) tyrosine kinase inhibitor (TKI), causes cell growth arrest in the G1-phase and induces apoptosis in a variety of tumor cells [12]. The pivotal BR.21 study showed that erlotinib brought a survival benefit with delayed progression of symptoms and improvement of QOL in a wide range of NSCLC patients, irrespective of *EGFR* mutation status [8]. Erlotinib became available in Japan for the treatment of relapsed NSCLC at an approved daily dose of 150 mg in October 2007.

Pemetrexed and erlotinib have advantages over docetaxel in that they have a better toxicity profile and more favorable tolerability. Since these two agents have different mechanisms of action and minimum overlap toxicities, their combination is expected to offer synergistic antitumor efficacy without increased toxicity. However, based on preclinical *in vitro* findings, careful attention should be paid to the combined administration schedule for pemetrexed and erlotinib. It was found that, when human NSCLC cells were exposed to pemetrexed followed by erlotinib, erlotinib synergistically potentiated the cytotoxic effect of pemetrexed [13,14]. This cytotoxic synergism was observed in both erlotinib-sensitive and -resistant cell lines. In this order of administration, pemetrexed induced cells to accumulate in the M-phase where erlotinib is most cytotoxic. Hence, this sequential combination enhances antitumor activity. In contrast, when NSCLC cells were treated with these agents in reverse order, antagonistic interaction was observed. This was due to the fact that erlotinib induced G1 arrest, resulting in a reduction in the number of cell entering the S-phase, the crucial cell cycle phase for the exertion of pemetrexed-mediated cytotoxicity [13,14]. A similar finding has been reported for the combination of erlotinib with docetaxel [15].

Assessment of treatment-related adverse events (AEs) associated with the pemetrexed-erlotinib combination is important for future clinical application, side by side with evaluation of the expected additive antitumor effects. EGFR-TKIs have different toxicity profiles between Asians and Caucasians. EGFR-TKI-induced interstitial lung disease is observed more frequently in Asians, especially in the Japanese. Increased hematologic toxicities have been reported in a recent phase I study of combination therapy involving gefitinib and vinorelbine [16]. Therefore, a safety evaluation in Japanese patients will inevitably be required for the combination of EGFR-TKI with cytotoxic drugs. Hence, we conducted a phase I trial to determine the dose-limiting toxicity (DLT) and to establish a recommended dose (RD), by estimating the maximum tolerated dose (MTD) of the combination of pemetrexed and intermittent erlotinib in a second-line setting for previously treated Japanese patients with advanced NSCLC.

## Methods

### Patient selection

The following eligibility criteria were mandatory for patient enrollment: (1) histologically or cytologically confirmed stage IIIB/IV nonsquamous NSCLC which had progressed on or after first-line platinum-based chemotherapy; (2) age ≥ 20 years; (3) measurable disease according to Response Evaluation Criteria in Solid Tumors (RECIST) version 1.1; (4) an Eastern Cooperative Oncology Group (ECOG) performance status (PS) grade of 0–1; (5) adequate hematologic (absolute white blood cell count ≥ 3000/μL, neutrophil count ≥ 1500/μL, platelet count ≥ 100000/μL and hemoglobin ≥ 9.0 g/dL), renal (serum creatinine ≤ 1.5 times the institutional upper limit of normal [ULN]), liver (serum bilirubin ≤ 1.5 mg/dL and aminotransferases ≤ 2.0 times that of the ULN), and respiratory (SpO_2_ ≥ 90% under room air) functions; (6) estimated life expectancy of more than 3 months; and (7) provision of written informed consent. Patients with asymptomatic brain metastases were also eligible. On the other hand, patients who had previously received HER-targeted therapy or pemetrexed were excluded. Patients with clinically significant ophthalmologic abnormalities or other disorders that might increase the risk of corneal epithelial injury were also excluded. In addition, patients who were pregnant or had unstable medical conditions were excluded. Patients with childbearing potential were required to use a medically acceptable contraceptive. Also considered ineligible were patients that had undergone invasive therapeutic procedures including pleurodesis and intrathoracic drainage within 2 weeks, chemotherapy within 3 weeks, thoracic radiotherapy within 12 weeks or extra-thoracic radiotherapy within 2 weeks.

### Study design and treatment plan

This open-label phase I dose-finding study evaluated the safety dose of erlotinib in combination with pemetrexed for a future phase II study. The primary objective was to determine the RD of erlotinib combined with the fixed dose (500 mg/m^2^) of pemetrexed and the DLTs. The secondary objective was to evaluate the safety profile. The study was carried out in Osaka Police Hospital and Osaka University Hospital after the protocol was approved by each institutional review board. The study protocol adhered to the principles outlined in the Guideline for Good Clinical Practice and Declaration of Helsinki and the trial was registered as UMIN000002900. Written informed consent was obtained from all patients before commencement of the study.

Patients were scheduled to receive 500 mg/m^2^ of pemetrexed intravenously on day 1, and erlotinib at a dose of 100 mg at Level 1 and 150 mg at Level 2, orally from days 2–16. Patients were also supplemented with 1 mg of vitamin B_12_ intramuscularly every 9 weeks and a daily dose of 500 μg of folic acid orally. The treatment cycle was repeated every 21 days with permission of delay up to 14 days until the first appearance of unacceptable toxicity, disease progression or death. The dose escalation followed the standard ‘3 + 3’ rule. When none of the first three patients experienced a DLT at the Level 1 dose, three patients were treated at the Level 2 dose. If at least one of three of the first cohort in each level encountered a DLT, an additional cohort of three patients was evaluated at the same dose. No intrapatient dose escalation was permitted.

### Definition of DLT and MTD

DLTs were defined in advance as any of the following drug-related toxicities that occurred within the first two cycles: (1) grade 4 neutropenia lasting 7 days and longer; (2) febrile neutropenia lasting 7 days and longer; (3) grade 4 thrombocytopenia or requirement for platelet transfusion; (4) delay in the start of the next course of longer than 2 weeks due to prolonged toxicity; (5) grade 3 or 4 non-hematologic toxicity excluding hyponatremia, skin rash, nausea and vomiting; (6) necessity for more than one dose reduction in pemetrexed or erlotinib. The dose with which more than one third of patients experienced any DLT was considered as the MTD. The available maximum doses approved by health insurance in Japan were 150 mg for erlotinib and 500 mg/m^2^ for pemetrexed. The purpose of this study was to examine if erlotinib could enhance the antitumor activity of pemetrexed. Moreover, erlotinib beyond a dose of 150 mg is known to increase the incidence of severe AEs that would probably interfere with the administration of pemetrexed. For these reasons, dose escalation beyond the approved maximum doses was not planned even if they did not reach the MTD.

### Patient assessments

Patients underwent weekly physical examination and assessment of the following parameters: vital signs, PS, hematology and biochemistry. Before each cycle, the patient’s medical history was reviewed. All AEs and DLTs were reported throughout the study. Toxicity was graded according to the National Cancer Institute Common Terminology Criteria for AEs (CTCAE) version 3.0. Although not the primary endpoint of this phase I study, treatment efficacy was also assessed in accordance with RECIST version 1.1 and the best overall response was evaluated. Tumor size was calculated using computed tomography images at time points within 4 weeks before, and 4 and 8 weeks after initiation of the therapy protocol, and thereafter every 8 weeks until disease progression was confirmed.

## Results

### Patient characteristics

Twelve patients (Level 1, n = 6; Level 2, n = 6) were enrolled from two institutes between August 2009 and March 2011. The antitumor efficacy and RD data were finalized at the end of May 2011. All patients were also assessable for AEs, DLTs and best overall responses. The baseline characteristics of the nine male and three female patients (Table 1) where: median age 66 years (range, 48 to 78 years); PS grade 1 (nine cases); stage IV disease (nine cases); and one current and eight ex-smokers. Histologic types included seven adenocarcinomas, one large cell carcinoma and four poorly differentiated NSCLCs without the components of squamous cell carcinoma. *EGFR* mutations were also examined using the peptide nucleic acid-locked nucleic acid PCR clamp method in all patients. Two had activating point mutations G719S in exon 18 and L858R in exon 21.

**Table 1:** Patient characteristics (n = 12)

Median age, yrs. (range)	66 (48 – 78)
Gender	
Male/Female	9/3
PS	
0/1	3/9
Histology	
Adeno/Large/Other	7/1/4
Clinical Stage	
IIIB/IV	3/9
Smoking History	
Current/Ex/Never	1/8/3
EGFR mutation	
Positive/Negative	2/10
Best response to prior chemo	
PR/SD/PD/NE	3/5/3/1

### Safety, DLT and RD

Almost all patients experienced AEs to some extent regardless of causality and severity. As summarized in Table 2, dermal, gastrointestinal and hematologic disorders were frequently observed AEs. Severe AEs with grade 3/4 were neutropenia/leukopenia (n = 2), diarrhea (n = 1), skin rash (n = 1), pruritus (n = 2) and alanine aminotransferase (ALT) elevation (n = 1). At Level 1, one patient in the first cohort (n = 3) developed a DLT of grade 3 ALT elevation. Another patient in the second cohort (n = 3) experienced a DLT of repetitive grade 4 neutropenia that required a successive dose reduction of pemetrexed and termination of the treatment protocol. At Level 2, one patient in the first cohort (n = 3) developed a DLT of grade 3 diarrhea, which was probably caused by erlotinib, because it did not recur during post-protocol pemetrexed monotherapy. No patient in the second cohort (n = 3) experienced DLTs. In total, three out of 12 patients, two at Level 1 and one at Level 2, experienced DLTs. The MTD, defined as the dose at which more than one third of patients experience DLTs, was not reached. Finally, the RD for erlotinib was fixed at 150 mg.

**Table 2:** Adverse events in patients with advanced non-small cell lung cancer treated with erlotinib and pemetrexed

	**Level 1 (n = 6)**				**Level 2 (n = 6)**			
Hematologic	G1	G2	G3	G4	G1	G2	G3	G4
Neutropenia/Leukopenia	2	0	0	1	0	0	0	1
Thrombocytopenia	2	1	0	0	1	1	0	0
Anemia	3	3	0	0	4	1	0	0
Non-Hematologic	G1	G2	G3	G4	G1	G2	G3	G4
Nausea	3	0	0	0	2	1	0	0
Vomit	1	0	0	0	0	0	0	0
Anorexia	3	1	0	0	2	1	0	0
Malaise	1	1	0	0	0	0	0	0
Fatigue	2	1	0	0	3	0	0	0
Diarrhea	1	0	0	0	1	0	1	0
Constipation	1	0	0	0	2	0	0	0
Hiccup	0	0	0	0	0	1	0	0
Oral mucositis	1	0	0	0	3	0	0	0
Rash	2	2	1	0	1	5	0	0
Pruritus	1	0	1	0	2	0	1	0
Paronychia	0	0	0	0	2	0	0	0
Alopecia	0	0	0	0	1	0	0	0
Peripheral Edema	0	0	0	0	0	1	0	0
Alb	4	1	0	0	1	2	0	0
AST	4	2	0	0	3	1	0	0
ALT	0	3	1	0	2	2	0	0
ALP	2	0	0	0	2	1	0	0
Bilirubin	1	0	0	0	2	1	0	0
Na	0	0	0	0	3	0	0	0
K	2	0	0	0	1	0	0	0

### Causes for discontinuation of protocol treatment

Eight patients had discontinued the treatment protocol by the time of analysis. The reasons for withdrawal were disease progression (n = 3), DLT (n = 1), patient’s refusal to continue treatment (n = 2) and physician’s decision to terminate treatment (n = 2). Four patients are still on the treatment protocol. The median number of treatment courses was 3.5 (range, 1 to 15). Among the three DLTs, repetitive grade 4 neutropenia requiring a second dose reduction of pemetrexed automatically meant the termination of the study protocol. The DLT of grade 3 ALT elevation was thought to have been caused by erlotinib and on the basis of a decision made by a physician the patient was withdrawn from the study. However, the DLT recurred during a following post-protocol course of pemetrexed monotherapy at a dose of 500 mg/m^2^, and was thereafter ameliorated by reducing the dose to 375 mg/m^2^. We concluded that this AE was attributable to pemetrexed, and not to erlotinib. The patient who experienced the other DLT of grade 3 diarrhea refused to continue the therapy, although the DLT had improved to grade 2 and the protocol permitted a resumption of the therapy with a reduced dose of erlotinib (100 mg) and pemetrexed (375 mg/m^2^) in the next cycle. One patient at Level 1 was withdrawn from the study due to a physician’s decision, on the grounds that cancer-related pain caused by invasion of the primary tumor into the vertebrae had gradually worsened, while tumors had maintained stable disease (SD) in line with RECIST after nine cycles of treatment. One patient in Level 2 refused to continue the therapy after three cycles because of grade 2 peripheral edema.

### Antitumor efficacy

All patients were evaluated for response to the treatment protocol. Three achieved a partial response (PR), five maintained SD, three had disease progression and one, who was withdrawn from the study because of diarrhea in the first cycle, was not evaluable (NE). Thus, the best overall response rate (RR) and disease control rate (DCR) were 25.0% and 66.7%, respectively. Among the 10 patients without the activating *EGFR* mutation, three (30.0%) achieved a PR and three (30.0%) had SD. On the other hand, both of the two *EGFR* mutation-positive patients maintained SD. One with G719S in exon 18 maintained SD for longer than 6 months. The other with L858R in exon 21 showed a remarkable shrinkage in the shorter axis of the measurable tumor, but did not meet the PR criteria defined by the longest axis, and the disease was also stable for longer than 6 months.

## Discussion

Two phase I clinical trials that have investigated the safety and efficacy of the erlotinib-pemetrexed combination have been reported. In the first study by Ranson et al. [17], where both agents were started on the same day, DLTs were not experienced at doses of up to 150 mg/day of erlotinib plus 600 mg/m^2^ of pemetrexed; the RD was determined as being equal to each of the licensed single-agent doses (150 mg daily and 500 mg/m^2^ every 21 days, respectively). In the second study by Davies et al. [18], the RD and administration schedule was fixed at a dose of 250 mg from day 2 to 16 for erlotinib and 500 mg/m^2^ on day 1 for pemetrexed every 21 days. We used the same RD as in the first trial with an identical administration schedule to that used in the second trial, and have now moved to an ongoing phase II trial.

As for the safety profile, observed AEs were consistent with those reported in the former two phase I studies [17,18]. The most frequent AE was skin/dermal disorder, which was compatible with that reported in the previous studies of erlotinib monotherapy [19,20]. Other major AEs were hematologic disorders, ALT elevation and gastrointestinal disorders, which were commonly observed in monotherapy involving either agent. However, the majority of them were grade 1/2 and controllable. Additive toxicities of erlotinib and pemetrexed administered in combination seemed to be marginal compared with those observed in previous studies of either agent alone [11,19,20]. No patient experienced interstitial lung disease. Consequently, administrating intermittent erlotinib with pemetrexed was shown to be safe and tolerable.

The latest randomized phase II study involving pretreated nonsquamous NSCLC patients demonstrated that, as compared with pemetrexed (500 mg/m^2^, day 1) alone, combination of pemetrexed (500 mg/m^2^, day 1) with daily erlotinib (150 mg) significantly improved both median progression-free survival (3.2 months *vs*. 2.9 months; *p* <0.01) and overall survival (11.8 months *vs*. 7.8 months, *p* = 0.019), although the RR (17.1% *vs*. 10.8%) and DCR (55.8% *vs*. 51.8%) did not differ between the single and combined treatments [21]. Even though the design of this study differed from ours in that the erlotinib administration schedule was daily rather than intermittent, and that collection of *EGFR* mutation status data was discretionary rather than mandatory, the significant survival benefit brought about by this combination therapy has encouraged us to continue our ongoing phase II study until we have overall survival data.

When evaluated on the basis of RECIST two *EGFR*-mutated patients did not respond in our study. However, one patient with a L858R mutation in exon 21 practically achieved a PR. The other with a G719S mutation in exon 18 succeeded in maintaining a long SD, which was also practically beneficial, considering that in patients with the G719X mutation the reported RR to EGFR-TKIs was approximately 56% [22]. Collectively, the erlotinib and pemetrexed combination is not only well-tolerated, but also attractive in terms of antitumor efficacy, regardless of *EGFR* mutation status.

However, our study had a number of limitations. First, pharmacokinetic analysis of the two agents was not conducted and potential interactions were not investigated. Second, QOL was not assessed by questionnaire. QOL assessment would have been helpful in understanding the impact of AEs, since there was a high rate of them even though most were grade 1 or 2. These issues should be addressed in future trials.

Recently, maintenance therapy following platinum-doublet front-line therapy has been approved, as long as the treatment regimen has tolerable and noncumulative toxicity profiles. Pemetrexed and erlotinib have been shown to be effective and tolerable when used as single-agent maintenance therapy [23,24], and were approved as suitable agents for this purpose by the US-Food and Drug Administration in 2009 and the European Medicines Agency in 2010. Since we have now demonstrated the safety and possible efficacy of the combined use of these two agents in the current phase I study, it would be very interesting to compare the therapeutic efficacy of this combination with that of the two agents administered separately in a maintenance setting in the near future; no studies of this design have been reported to date.

In conclusion, the present study indicated that intermittent erlotinib (150 mg on days 2 to 16) in combination with pemetrexed (500 mg/m^2^ on day 1) when administered every 21 days is a feasible and well-tolerated regimen. A multi-centered, single-arm phase II study is currently ongoing to evaluate the efficacy and safety of this combination regimen.

## Conclusions

In the present study, combination chemotherapy with intermittent erlotinib and pemetrexed was found to be safe and well-tolerated. The combination of these agents could have additive antitumor effects against pretreated advanced nonsquamous NSCLC. Further investigation to substantiate the antitumor efficacy and safety of this combination regimen in a higher number of patients is currently ongoing in a phase II trial.

## Competing interests

The authors declare that they have no competing interests.

## Authors’ contributions

TK and KK conceived the study design. SM, TK, RT, HK, TN, MH, YT, IN and SY contributed to patient recruitment and carried out clinical study procedures. SM, TK and RT analyzed and interpreted the data. SM and TK prepared the manuscript. IT, KK and IK critically revised the manuscript for important intellectual content. All authors read and approved the final manuscript.

## Pre-publication history

The pre-publication history for this paper can be accessed here:

http://www.biomedcentral.com/1471-2407/12/296/prepub

## References

[B1] Jemal A Siegel R Xu J Ward E Cancer statistics, 2010 CA Cancer J Clin 2010 60 5 277 300 10.3322/caac.20073 20610543

[B2] Azzoli CG Baker S Temin S Pao W Aliff T Brahmer J Johnson DH Laskin JL Masters G Milton D American Society of Clinical Oncology Clinical Practice Guideline update on chemotherapy for stage IV non-small-cell lung cancer J Clin Oncol 2009 27 36 6251 6266 10.1200/JCO.2009.23.5622 19917871 PMC2793036

[B3] Reck M von Pawel J Zatloukal P Ramlau R Gorbounova V Hirsh V Leighl N Mezger J Archer V Moore N Phase III trial of cisplatin plus gemcitabine with either placebo or bevacizumab as first-line therapy for nonsquamous non-small-cell lung cancer: AVAil J Clin Oncol 2009 27 8 1227 1234 10.1200/JCO.2007.14.5466 19188680

[B4] Sandler A Gray R Perry MC Brahmer J Schiller JH Dowlati A Lilenbaum R Johnson DH Paclitaxel-carboplatin alone or with bevacizumab for non-small-cell lung cancer N Engl J Med 2006 355 24 2542 2550 10.1056/NEJMoa061884 17167137

[B5] Scagliotti GV Parikh P von Pawel J Biesma B Vansteenkiste J Manegold C Serwatowski P Gatzemeier U Digumarti R Zukin M Phase III study comparing cisplatin plus gemcitabine with cisplatin plus pemetrexed in chemotherapy-naive patients with advanced-stage non-small-cell lung cancer J Clin Oncol 2008 26 21 3543 3551 10.1200/JCO.2007.15.0375 18506025

[B6] Hanna N Shepherd FA Fossella FV Pereira JR De Marinis F von Pawel J Gatzemeier U Tsao TC Pless M Muller T Randomized phase III trial of pemetrexed versus docetaxel in patients with non-small-cell lung cancer previously treated with chemotherapy J Clin Oncol 2004 22 9 1589 1597 10.1200/JCO.2004.08.163 15117980

[B7] Shepherd FA Dancey J Ramlau R Mattson K Gralla R O'Rourke M Levitan N Gressot L Vincent M Burkes R Prospective randomized trial of docetaxel versus best supportive care in patients with non-small-cell lung cancer previously treated with platinum-based chemotherapy J Clin Oncol 2000 18 10 2095 2103 10811675 10.1200/JCO.2000.18.10.2095

[B8] Shepherd FA Rodrigues Pereira J Ciuleanu T Tan EH Hirsh V Thongprasert S Campos D Maoleekoonpiroj S Smylie M Martins R Erlotinib in previously treated non-small-cell lung cancer N Engl J Med 2005 353 2 123 132 10.1056/NEJMoa050753 16014882

[B9] Hanauske AR Chen V Paoletti P Niyikiza C Pemetrexed disodium: a novel antifolate clinically active against multiple solid tumors Oncologist 2001 6 4 363 373 10.1634/theoncologist.6-4-363 11524555

[B10] Cullen MH Zatloukal P Sorenson S Novello S Fischer JR Joy AA Zereu M Peterson P Visseren-Grul CM Iscoe N A randomized phase III trial comparing standard and high-dose pemetrexed as second-line treatment in patients with locally advanced or metastatic non-small-cell lung cancer Ann Oncol 2008 19 5 939 945 10.1093/annonc/mdm592 18283036

[B11] Ohe Y Ichinose Y Nakagawa K Tamura T Kubota K Yamamoto N Adachi S Nambu Y Fujimoto T Nishiwaki Y Efficacy and safety of two doses of pemetrexed supplemented with folic acid and vitamin B12 in previously treated patients with non-small cell lung cancer Clin Cancer Res 2008 14 13 4206 4212 10.1158/1078-0432.CCR-07-5143 18594001

[B12] Dancey J Sausville EA Issues and progress with protein kinase inhibitors for cancer treatment Nat Rev Drug Discov 2003 2 4 296 313 10.1038/nrd1066 12669029

[B13] Giovannetti E Lemos C Tekle C Smid K Nannizzi S Rodriguez JA Ricciardi S Danesi R Giaccone G Peters GJ Molecular mechanisms underlying the synergistic interaction of erlotinib, an epidermal growth factor receptor tyrosine kinase inhibitor, with the multitargeted antifolate pemetrexed in non-small-cell lung cancer cells Mol Pharmacol 2008 73 4 1290 1300 10.1124/mol.107.042382 18187583

[B14] Li T Ling YH Goldman ID Perez-Soler R Schedule-dependent cytotoxic synergism of pemetrexed and erlotinib in human non-small cell lung cancer cells Clin Cancer Res 2007 13 11 3413 3422 10.1158/1078-0432.CCR-06-2923 17545550

[B15] Furugaki K Iwai T Shirane M Kondoh K Moriya Y Mori K Schedule-dependent antitumor activity of the combination with erlotinib and docetaxel in human non-small cell lung cancer cells with EGFR mutation, KRAS mutation or both wild-type EGFR and KRAS Oncol Rep 2010 24 5 1141 1146 20878103 10.3892/or_00000965

[B16] Yoshimura M Nakamura S Imamura F Ueno K Yamamoto S Igarashi T Severe myelotoxicity in a combination of gefitinib and vinorelbine Lung Cancer 2004 45 1 121 123 10.1016/j.lungcan.2004.01.006 15196742

[B17] Ranson M Reck M Anthoney A Hanauske AR Dean E Melezinek I Klingelschmitt G Kletzl H Blatter J Twelves C Erlotinib in combination with pemetrexed for patients with advanced non-small-cell lung cancer (NSCLC): a phase I dose-finding study Ann Oncol 2010 21 11 2233 2239 10.1093/annonc/mdq246 20444843

[B18] Davies AM Ho C Beckett L Lau D Scudder SA Lara PN Perkins N Gandara DR Intermittent erlotinib in combination with pemetrexed: phase I schedules designed to achieve pharmacodynamic separation J Thorac Oncol 2009 4 7 862 868 10.1097/JTO.0b013e3181a94b08 19494788

[B19] Kubota K Nishiwaki Y Tamura T Nakagawa K Matsui K Watanabe K Hida T Kawahara M Katakami N Takeda K Efficacy and safety of erlotinib monotherapy for Japanese patients with advanced non-small cell lung cancer: a phase II study J Thorac Oncol 2008 3 12 1439 1445 10.1097/JTO.0b013e31818d6702 19057270

[B20] Takahashi T Yamamoto N Nukiwa T Mori K Tsuboi M Horai T Masuda N Eguchi K Mitsudomi T Yokota S Phase II study of erlotinib in Japanese patients with advanced non-small cell lung cancer Anticancer Res 2010 30 2 557 563 20332470

[B21] von Pawel J Papai-Szekely Z Vinolas N Sederholm C Klima M Desaiah D Leschinger MI Dittrich C A randomized phase II study of pemetrexed versus pemetrexed plus erlotinib in second-line treatment for locally advanced or metastatic, nonsquamous NSCLC [abstract] J Clin Oncol 2011 29 s7526 10.1016/j.ejca.2014.03.007 24703574

[B22] Mitsudomi T Yatabe Y Mutations of the epidermal growth factor receptor gene and related genes as determinants of epidermal growth factor receptor tyrosine kinase inhibitors sensitivity in lung cancer Cancer Sci 2007 98 12 1817 1824 10.1111/j.1349-7006.2007.00607.x 17888036 PMC11159145

[B23] Cappuzzo F Ciuleanu T Stelmakh L Cicenas S Szczesna A Juhasz E Esteban E Molinier O Brugger W Melezinek I Erlotinib as maintenance treatment in advanced non-small-cell lung cancer: a multicentre, randomised, placebo-controlled phase 3 study Lancet Oncol 2010 11 6 521 529 10.1016/S1470-2045(10)70112-1 20493771

[B24] Ciuleanu T Brodowicz T Zielinski C Kim JH Krzakowski M Laack E Wu YL Bover I Begbie S Tzekova V Maintenance pemetrexed plus best supportive care versus placebo plus best supportive care for non-small-cell lung cancer: a randomised, double-blind, phase 3 study Lancet 2009 374 9699 1432 1440 10.1016/S0140-6736(09)61497-5 19767093

